# Systemic Hypoxia Increases Retinal Blood Flow but Reduces the Oxygen Saturation Less in Peripheral Than in Macular Vessels in Normal Persons

**DOI:** 10.1167/iovs.66.6.43

**Published:** 2025-06-13

**Authors:** Jacob Drachmann, Line Petersen, Signe Krejberg Jeppesen, Toke Bek

**Affiliations:** 1 Department of Ophthalmology, Aarhus University Hospital, Aarhus, Denmark

**Keywords:** retinal autoregulation, blood flow, regional variation, retinal oximetry, Doppler OCT

## Abstract

**Purpose:**

Retinal vascular diseases are characterized by regional differences in the distribution of morphological lesions that may be related to regional differences in the autoregulation of retinal blood flow. The purpose of the present study was to investigate how systemic hypoxia affects blood flow and oxygen saturation in different vascular areas in normal persons.

**Methods:**

In 28 normal persons, oxygen saturation and vessel diameters were measured by dual wavelength retinal oximetry, and blood flow velocity by Doppler OCT in the peripapillary arterioles supplying and venules draining the four retinal quadrants, and in an arteriolar and a venular branch from the upper temporal arcade towards respectively the retinal periphery and the macular area. The measurements were performed during normoxia and during breathing of a gas mixture containing 12.5% oxygen.

**Results:**

Systemic hypoxia reduced the oxygen saturation in peripapillary arterioles by approximately 11% (*P* < 0.001) and increased the peripapillary blood flow by approximately 40% (*P* < 0.001). Systemic hypoxia also reduced the oxygen saturation in the macular arterioles and venules and in the peripheral arterioles (*P* < 0.003), but not in the peripheral venules (*P* = 95). The blood flow increased significantly in both macular and peripheral arterioles and venules (*P* < 0.04).

**Conclusions:**

Systemic hypoxia can affect the venous oxygen saturation in vessels draining the retinal periphery and the macular area differently. The findings may help to understand the regionally varying manifestations of retinal vascular disease.

Retinal vascular diseases exhibit a characteristic morphological pattern with hemorrhages, exudates, and edema caused by hyperperfusion in the macular area^1^ and capillary occlusion leading to ischemia and hypoxia in the retinal periphery.^2^^,^^3^ The mechanisms underlying these regional differences are unknown but may involve disturbances in the autoregulation of retinal blood flow.^4^^–^^6^ A further investigation of the pathophysiology underlying these characteristics requires an understanding of regional differences in retinal autoregulation under normal conditions.

A previous study has shown that an increase in the arterial blood pressure induced by isometric exercise in normal persons can increase the oxygen saturation in venules draining the retinal periphery but not in venules draining the macular area.^7^ This can be explained as a consequence of increased peripheral blood flow caused by either dilation of the capillary bed or shunting of the blood to bypass the microcirculation.^8^ The finding therefore suggests that pressure autoregulation in the retinal periphery can be less efficient than in the macular area.

Another mechanism for regulating blood flow is metabolic autoregulation where hypoxia induced by increased metabolism leads to vasodilation to increase blood flow and normalize the oxygen saturation.^9^^–^^11^ However, regional differences in oxygen saturation and blood flow secondary to retinal hypoxia have not been studied in detail in vivo.

Therefore in the present study changes in oxygen saturation, diameter and blood flow were studied in peripapillary, peripheral and macular arterioles and venules in 28 normal persons breathing ambient air. The examinations were subsequently repeated during systemic hypoxia induced by breathing a gas mixture containing 12.5% oxygen.

## Material and Methods

### Participants

Twenty-eight healthy persons (12 men and 16 women) aged 19 to 38 years (mean ± SD = 28.1 ± 3.5 years) with no known previous or current systemic or ocular diseases were recruited by announcement among students and employees at Aarhus University Hospital. The respondents who showed interest in participation were informed about the study orally and in writing, and were given at least 24 hours before acceptance and final consent for participation. The participants were instructed to avoid consumption of caffeine^12^ and alcohol^13^ 12 hours before the examination.

The study had been approved by The Regional Committee for Scientific Ethics and The Danish Medicines Agency and was conducted in accordance with the declaration of Helsinki.

### Examination

#### Ophthalmologic Examination

A routine ophthalmologic examination was performed to exclude participants with ocular disease. The examination included measurement of best-corrected visual acuity in accordance with the standards of the Early Treatment Diabetic Retinopathy Study.^14^

Mydriasis was achieved with phenylephrine 10% (Skanderborg Pharmacy, Skanderborg, Denmark) and tropicamide 1% (Bausch & Lomb, Surrey, UK), followed by slit lamp examination and 60° fundus photography (Canon CF 60Z; Canon, Amstelveen, Netherlands) centered on the fovea and the optic disc. The axial length of the eye was measured by optical biometry (Lenstar LS 900; Haag-Streit AG, Köniz, Switzerland). The baseline data of the participants are shown in Table 1.

**Table 1.** Demographic Data and Baseline Values of the Participants (Mean ± SD)

	Study Eye (16 Right/12 Left)
BCVA (ETDRS letters)	93.9 ± 2.9
Axial length (mm)	24.1 ± 1.3
Refraction (diopters spherical equivalent)	−1.4 ± 3.1

BCVA, best-corrected visual acuity; ETDRS, Early Treatment Diabetic Retinopathy Study.

#### Selection of Study Eye and Vessels

On the basis of the fundus photographs, the eye with the best fit to the following criteria was selected: A set of four branches from the upper temporal arcade consisting of an arteriole supplying and a venule draining, respectively, the macular area and the retinal periphery, each with a diameter of at least one third of the main vessel. If both eyes had an equal number of eligible vessel branches, the eye with the shortest distance between the distal and proximal branching of the four vessels from the arcade was selected. This resulted in the inclusion of vessels from 16 right eyes and 12 left eyes. Together with the upper and lower temporal and nasal arterioles and venules, this resulted in 12 vessels to be studied in each eye. On each of these vessels, a segment was delimited for the measurements. For the peripapillary vessels, the segment extended from the disk margin to one disk diameter distally. For the peripheral and macular branches, the segment extended from the branching point at the upper temporal vessel to one disk diameter distally from this point, either towards the fovea or towards the retinal periphery. This resulted in the identification of a complete set of 12 vessels in all participants except one, where a peripheral venular branch meeting the inclusion criteria could not be identified. This resulted in a total of 335 vessel segments to be studied.

#### Retinal Blood Velocity

Blood flow velocity was measured using a dual-beam Fourier-domain Doppler optical coherence tomography (Doppler OCT) system developed at the Center for Medical Physics and Biomedical Engineering in Vienna, Austria.^15^^,^^16^ The system uses two light beams that illuminate a cross-section of the retina at different angles. The system operates at a central wavelength of 839 nm, with a scan length of approximately 2 mm on the retinal plane and a frame rate of 9 B-scans per second. When the light interacts with moving red blood cells, it undergoes a phase shift, which can be translated into a linear velocity of the blood.

#### Retinal Oxygen Saturation

Retinal oxygen saturations were measured by a dual-wavelength oximeter (model T1; Oxymap, Reykjavik, Iceland), which is a fundus camera (Topcon TRC-50DX; Topcon Corporation, Tokyo, Japan) that captures images simultaneously at two different wavelengths (570 nm and 600 nm). Because of the different absorbance spectra of oxygenated and deoxygenated hemoglobin, the difference in light absorbance between the two images can be translated to a value for the oxygen saturation in the vessel.

### Examination Protocol

**Figure 1.**
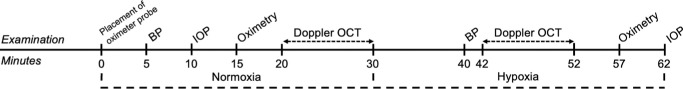
The examination protocol. BP, blood pressure; IOP, intraocular pressure.

The experimental protocol is shown in Figure 1. Before the examination, the probe from a pulse oximeter (Nellcor OxiMax N-65; Medtronic, Minneapolis, MN, USA) was placed on the left middle finger to allow measurements of the systemic oxygen saturation throughout the examination.

Preliminary experiments had shown that two minutes after a participant had moved to a new measuring device, the Mean arterial pressure (MAP) had always stabilized to be within 5 mm Hg and the systemic oxygen saturation to be within 2% of the initial recording. Therefore all resting periods during the experiments were at least two minutes, and to limit the duration of the experiment never exceeded five minutes.

#### Normoxia

The following steps were performed while breathing ambient air:

1. The participant was seated in a chair to rest for five minutes, after which the baseline blood pressure and pulse rate were measured on the upper left arm using an automated oscillometric monitor (Omron 705IT; Omron Healthcare, Kyoto, Japan) and the systemic oxygen saturation was recorded.
2. The participant was moved to the non-contact tonometer (Tonoref II; Nidek, Gamagori, Aichi, Japan) and after a resting period, the intraocular pressure was measured and the systemic oxygen saturation was recorded.
3. The participant was moved to the oximeter, and after a resting period, oximetry images were captured with centering on the fovea, the optic disc and the upper and lower temporal vascular arcades.^17^ The procedure was followed by a recording of the systemic oxygen saturation.
4. The participant was moved to the Doppler OCT, and after a resting period, scanning was performed to measure blood flow. The scanning line could be oriented either horizontally or vertically, with the orientation set to obtain an angle between the scan and the vessel as close to perpendicular as possible. The scans were positioned to include the highest number of the selected vessel segments, thereby reducing the number of scans required. This resulted in (mean, range) 7.6, 4–10 scans per participant. When a scan only included one vessel, scanning was performed as proximally on the vessel segment as possible. The systemic oxygen saturation was recorded immediately after each new scan had been obtained.

#### Hypoxia

The participant remained seated in front of the Doppler OCT and began breathing a gas mixture containing 12.5% oxygen and 87.5% nitrogen through a flexible mouthpiece under non-rebreathing conditions. The mouthpiece was connected to a Mapleson C bagging system with a 2 L reservoir bag (Intersurgical, Berkshire, UK), and hypoxic breathing was continued until the end of the experiment. A nose clip was used to prevent the inspired air from mixing with ambient air.

5. After 10 minutes of breathing the hypoxic gas mixture, the blood pressure and pulse rate were measured and the systemic oxygen saturation was recorded. Hereafter, Doppler OCT scanning was performed with systemic oxygen saturation recordings as described in step 4.
6. The participant was moved to be seated in front of the oximeter and after a resting period, oximetry was repeated as described in step 3.
7. The participant was moved to the tonometer and after a resting period, the intraocular pressure was measured as described in step 2.

#### Adverse Effects

One participant experienced claustrophobia while wearing the breathing system during hypoxia, which resulted in the omission of recordings.

### Data Analysis

#### Doppler OCT

Recordings obtained during normoxia were obtained from the 335 selected vessel segments. Three vessel recordings from one examination obtained during hypoxia were omitted from the participant experiencing claustrophobia. Therefore recordings were available from the remaining 332 vessel segments. During the analysis, six normoxia and 18 hypoxia recordings were excluded because of unsteady fixation, and one normoxia and seven hypoxia recordings were excluded because the phase shift exceeded the measurement range that is confined to values between −π and π.^15^ The remaining 328 normoxia and 307 hypoxia recordings were analyzed using custom-developed software (DOCTstudio, version 0.9, 30.11.2016), which converted the phase shift of the backscattered light, resulting from its interaction with moving red blood cells, into a linear velocity of the blood. The software adjusted for the axial length of the eye, the deviation of the angle from perpendicular between the scanning line and the vessels and for the angle at which the two light beams illuminated the retinal plane.

#### Retinal Oximetry

The image centered on the optic disk was used to analyze the peripapillary vessels, and the image centered on the upper temporal vascular arcade to analyze the branches toward the macular area and the retinal periphery. The images were analyzed using the Oxymap Analyzer software (Oxymap Analyzer version: 2.5.2, v2), and the oxygen saturations were recorded as the average value along the length of the defined vessel segments.

The software calculated the oxygen saturation by determining the ratio between the light reflected from the vessels (I) and the perivascular retina (I_0_), defined as the optical density (OD = log(I/I_0_)), for the 570 nm and 600 nm wavelength images. These ratios were used to calculate the optical density ratio (ODR) as ODR = OD_600_/OD_570_. Subsequently, the oxygen saturation was calculated as SatO_2_ = (a * ODR + b) + (c * d + k), with a = −1.28, b = 1.24, c = 0.0097, k = −0.14, and d = the vessel diameter measured by the Oxymap software.^18^^,^^19^

#### Distance From the Optic Disc to the Branching Points

The distance along the upper temporal arcade from the disc margin to the branching points of the macular and peripheral branches were measured using the Oxymap software. There were no significant differences in this distance between arterioles (mean ± SD) (peripheral: 490.3 ± 293.0 pixels, macular: 378.8 ± 241.4 pixels, *P* = 0.12) or venules (peripheral: 594.6 ± 232.4 pixels, macular: 543.0 ± 353.4 pixels, *P* = 0.53). Pixel values multiplied by 9.3 corresponded to micrometers based on Gullstrand's standard eye.

#### Diameter Measurements

The 570 nm fundus photographs obtained by retinal oximetry were exported in JPG format and uploaded to the open-source MATLAB software Automated Retinal Image Analyzer (ARIA, V1-09-12-11). The software uses a vessel detection algorithm to define vessel borders and calculate vessel diameters.^20^ The diameter measurements were calculated as the mean of individual measurements taken for each pixel along each of the selected vessel segments.

#### Blood Flow

Blood flow (Q) was calculated as Q = (v * d^2^ * π) / 4, where v was the linear velocity obtained by Doppler OCT and d was the vessel diameter measured using the ARIA software.

#### Blood Pressure

MAP was calculated as MAP = 1/3 * BP_s_ + 2/3 * BP_d_, where BP_s_ was the systolic blood pressure and BP_d_ was the diastolic blood pressure.

#### Statistical Analysis

It has previously been shown that a sample size of 27 participants is required to detect changes in oxygen saturation and blood flow with an alpha-risk of 5% and a power of 80% with the techniques used in this study.^21^ All analyses were performed using the statistical package STATA (version 17.0; StataCorp, College Station, TX, USA). Normal distribution of data was confirmed with probability plots. Unpaired *t*-test was used to test if retinal oxygen saturation, vessel diameter, linear velocity and blood flow differed significantly between peripheral and macular arterioles and venules, and to test if the distance from the optic disc along the vessel to the branching point differed between macular and peripheral arterioles and venules. Paired *t*-test was used to test if systemic oxygen saturation, MAP, pulse rate, retinal oxygen saturation, vessel diameter, linear velocity and blood flow changed significantly from normoxia to hypoxia, to test if the oxygen saturation measured systemically was different from that measured in peripapillary arterioles, and to test whether the changes in oxygen saturation, vessel diameter, linear velocity and blood flow differed among peripheral and macular arterioles and venules. One-way ANOVA was used to test if the systemic oxygen saturation changed throughout the experimental protocol during normoxia or during hypoxia, and if the percentage change in retinal oxygen saturation, vessel diameter, linear velocity and blood flow from normoxia to hypoxia differed significantly among the peripapillary arterioles and venules, respectively.

## Results

Table 2 shows that breathing the hypoxic gas mixture significantly reduced the systemic oxygen saturation (*P* < 0.001) and increased the mean arterial blood pressure (*P* = 0.02) and the pulse rate (*P* < 0.001), but it had no significant effect on the intraocular pressure (*P* = 0.2). There were no significant changes in the continuous measurements of the systemic oxygen saturation throughout the examinations, neither during normoxia (*P* = 0.9) nor during hypoxia (*P* = 0.8).

**Table 2.** The Measured Parameters Determining Oxygen Delivery to the Eye During Normoxia and Hypoxia (Mean ± SD)

	Normoxia	Hypoxia	*P* Value
Systemic oxygen saturation	98.1% ± 1.2%	85.8% ± 3.8%	<0.001
Mean arterial blood pressure (mm Hg)	89.0 ± 5.7	92.4 ± 7.5	0.02
Pulse rate (beats/min)	65.7 ± 9.9	71.7 ± 11.1	<0.001
Intraocular pressure (mm Hg)	14.5 ± 2.6	14.8 ± 2.8	0.2

Table 3 shows that in all peripapillary arterioles and venules, systemic hypoxia significantly reduced the oxygen saturation (*P* < 0.02 for all comparisons) and increased the vessel diameter (*P* < 0.02 for all comparisons), the linear velocity (*P* < 0.03 for all comparisons) and the blood flow (*P* < 0.002 for all comparisons). There were no significant differences between the percentage changes of these parameters among the four peripapillary arterioles (*P* > 0.07 for all comparisons) or venules (*P* > 0.4 for all comparisons). Therefore the mean of the oxygen saturation, vessel diameter and linear velocity, and the sum of the blood flow in the four peripapillary branches of respectively arterioles and venules were used to represent the overall peripapillary values for these parameters during respectively normoxia and hypoxia. The sum of the measured blood flow in the four peripapillary arterioles was not significantly different from that in the four peripapillary venules, neither during normoxia (*P* = 0.2) nor during hypoxia (*P* = 0.06).

**Table 3.** Oxygen Saturation, Vessel Diameter, Linear Velocity, and Blood Flow in Peripapillary Vessels From Normoxia to Hypoxia (Mean ± SD)

Location/Vessel	Saturation	Diameter (Pixel)	Velocity (mm/s)	Blood Flow (µL/Min)
Upper temporal				
Arteriole				
Normoxia	94.0% ± 5.3%	14.4 ± 2.5	16.2 ± 5.2	14.2 ± 8.1
Hypoxia	83.4% ± 7.4%	15.4 ± 2.4	18.4 ± 5.9	18.3 ± 8.8
*P*	<0.001	0.002	0.009	<0.001
Venule				
Normoxia	56.0% ± 7.9%	18.7 ± 2.5	11.6 ± 3.2	17.2 ± 8.6
Hypoxia	52.0% ± 8.7%	20.0 ± 2.8	13.7 ± 4.4	23.4 ± 11.8
*P*	0.02	<0.001	0.007	<0.001
Upper nasal				
Arteriole				
Normoxia	94.1% ± 7.7%	12.2 ± 2.3	11.7 ± 4.6	7.0 ± 4.7
Hypoxia	85.3% ± 7.6%	12.8 ± 2.1	15.4 ± 5.4	10.5 ± 6.1
*P*	<0.001	0.02	0.003	<0.001
Venule				
Normoxia	57.1% ± 9.0%	14.3 ± 2.1	8.6 ± 2.2	7.3 ± 3.1
Hypoxia	53.7% ± 9.7%	15.7 ± 2.4	10.4 ± 3.7	10.7 ± 5.5
*P*	0.003	<0.001	0.01	<0.001
Lower temporal				
Arteriole				
Normoxia	89.8% ± 6.0%	14.6 ± 2.8	16.4 ± 5.3	13.2 ± 5.0
Hypoxia	76.6% ± 7.5%	15.7 ± 2.5	19.7 ± 6.2	18.5 ± 5.7
*P*	<0.001	0.003	0.02	<0.001
Venule				
Normoxia	50.5% ± 10.7%	19.3 ± 3.1	11.8 ± 2.2	17.8 ± 5.9
Hypoxia	44.7% ± 10.4%	20.6 ± 2.9	15.7 ± 4.6	26.1 ± 10.0
*P*	0.004	<0.001	<0.001	<0.001
Lower nasal				
Arteriole				
Normoxia	95.2% ± 4.8%	11.9 ± 2.4	12.3 ± 4.4	6.6 ± 3.9
Hypoxia	84.4% ± 8.3%	12.9 ± 2.3	15.0 ± 5.7	10.1 ± 6.5
*P*	<0.001	<0.001	0.009	0.001
Venule				
Normoxia	58.5% ± 9.4%	13.7 ± 2.3	7.4 ± 2.5	5.4 ± 3.0
Hypoxia	55.2% ± 9.7%	14.6 ± 2.4	9.4 ± 3.9	7.7 ± 4.1
*P*	0.01	<0.001	0.02	<0.001
Overall for the four peripapillary vessels	Average	Average	Average	Total
Arterioles				
Normoxia	93.3% ± 3.7%	13.3 ± 2.0	14.6 ± 3.5	39.1 ± 12.9
Hypoxia	82.5% ± 6.7%	14.2 ± 1.9	17.6 ± 3.7	53.7 ± 15.7
*P*	<0.001	<0.001	<0.001	<0.001
Venules				
Normoxia	55.5% ± 6.6%	16.5 ± 1.8	10.2 ± 1.6	44.5 ± 12.2
Hypoxia	51.4% ± 7.2%	17.7 ± 1.9	12.6 ± 3.0	65.6 ± 20.1
*P*	0.003	<0.001	<0.001	<0.001

The overall oxygen saturation measured in the peripapillary arterioles (mean ± SD) was significantly lower than the oxygen saturation measured in the systemic circulation during both normoxia (98.1% ± 1.2% vs. 93.3% ± 3.7%) and hypoxia (85.8% ± 3.8% vs. 82.5% ± 6.7%) (*P* < 0.002 for both comparisons). The reductions in the arterial oxygen saturation from normoxia to hypoxia were not significantly different between the measurements in the systemic circulation (−12.3% ± 3.8%) and in peripapillary (−10.8% ± 5.3%) arterioles (*P* = 0.2). However, the reduction in the oxygen saturation from normoxia to hypoxia was significantly smaller (*P* < 0.001) in the peripapillary venules (−4.1% ± 6.3%).

Table 4 shows that during normoxia the oxygen saturation was significantly higher in peripheral than in macular arterioles (*P* = 0.04) and significantly lower in peripheral than in macular venules (*P* < 0.001). Furthermore, peripheral venules had a significantly larger diameter and blood flow than macular venules (*P* < 0.05 for both comparisons), whereas none of the other studied parameters differed significantly between peripheral and macular arterioles and venules.

**Table 4.** The Oxygen Saturation, Vessel Diameter, Linear Velocity, and Blood Flow in Peripheral and Macular Arterioles and Venules During Normoxia (Mean ± SD)

Vessel/Location	Saturation	Diameter (Pixel)	Velocity (mm/s)	Blood flow (µL/Min)
Arterioles				
Peripheral	97.6% ± 4.7%	10.3 ± 2.0	8.5 ± 3.9	3.8 ± 2.6
Macular	94.7% ± 5.6%	10.7 ± 1.8	8.3 ± 4.0	3.9 ± 1.8
*P*	0.04	0.4	0.9	0.9
Venules				
Peripheral	63.3% ± 9.6%	12.4 ± 2.8	6.5 ± 2.4	4.3 ± 2.3
Macular	74.4% ± 6.4%	10.6 ± 2.4	6.6 ± 3.0	3.1 ± 1.9
*P*	<0.001	0.01	0.9	<0.05

**Figure 2.**
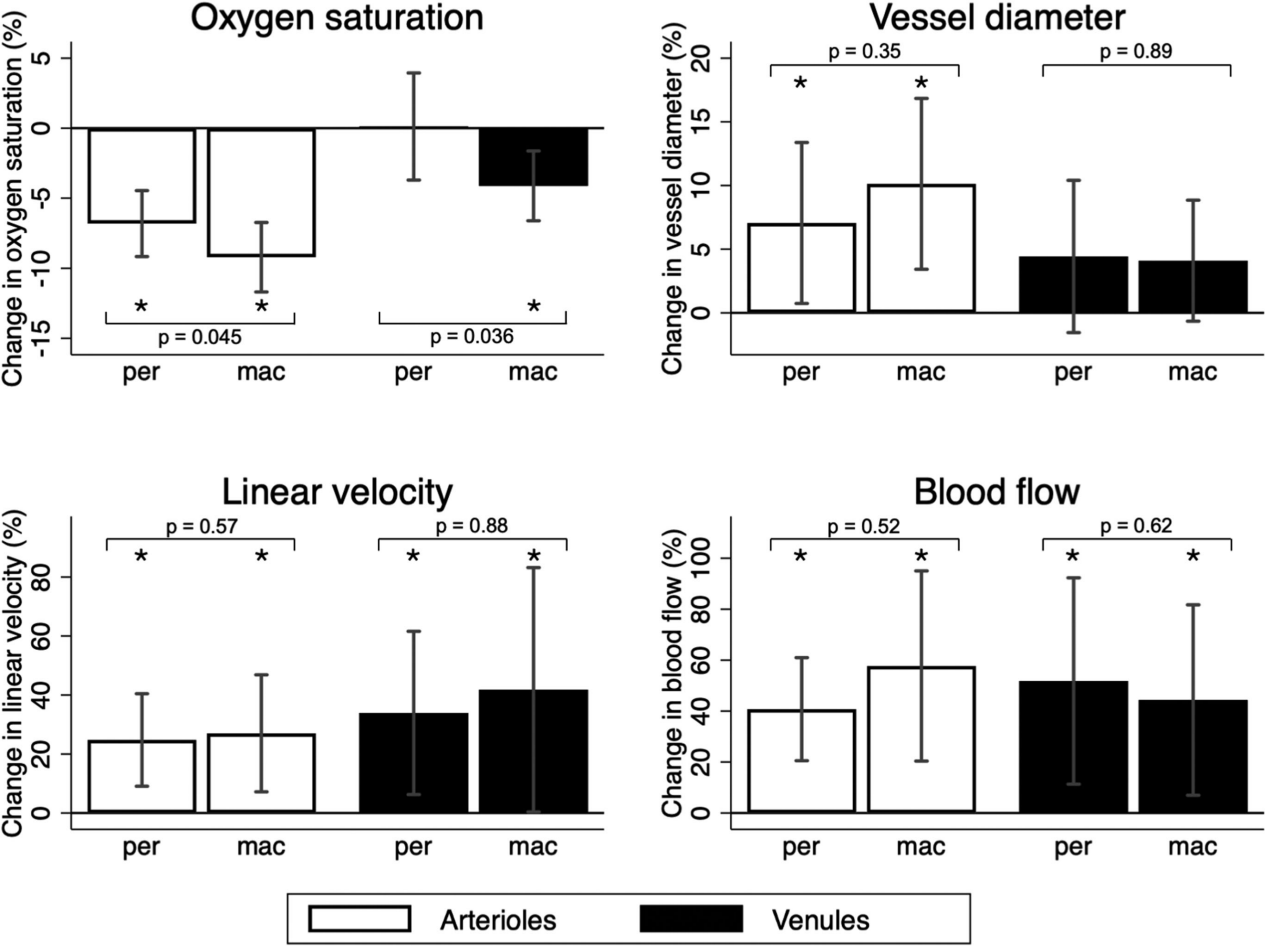
The percentage change in oxygen saturation, vessel diameter, linear velocity and blood flow in peripheral (per) and macular (mac) arterioles and venules from normoxia to hypoxia. Bars delimit the 95% confidence interval. *Asterisks* indicate a significant (*P* < 0.05) change from zero. *P* values refer to comparisons between the peripheral and macular responses.

**Figure 3.**
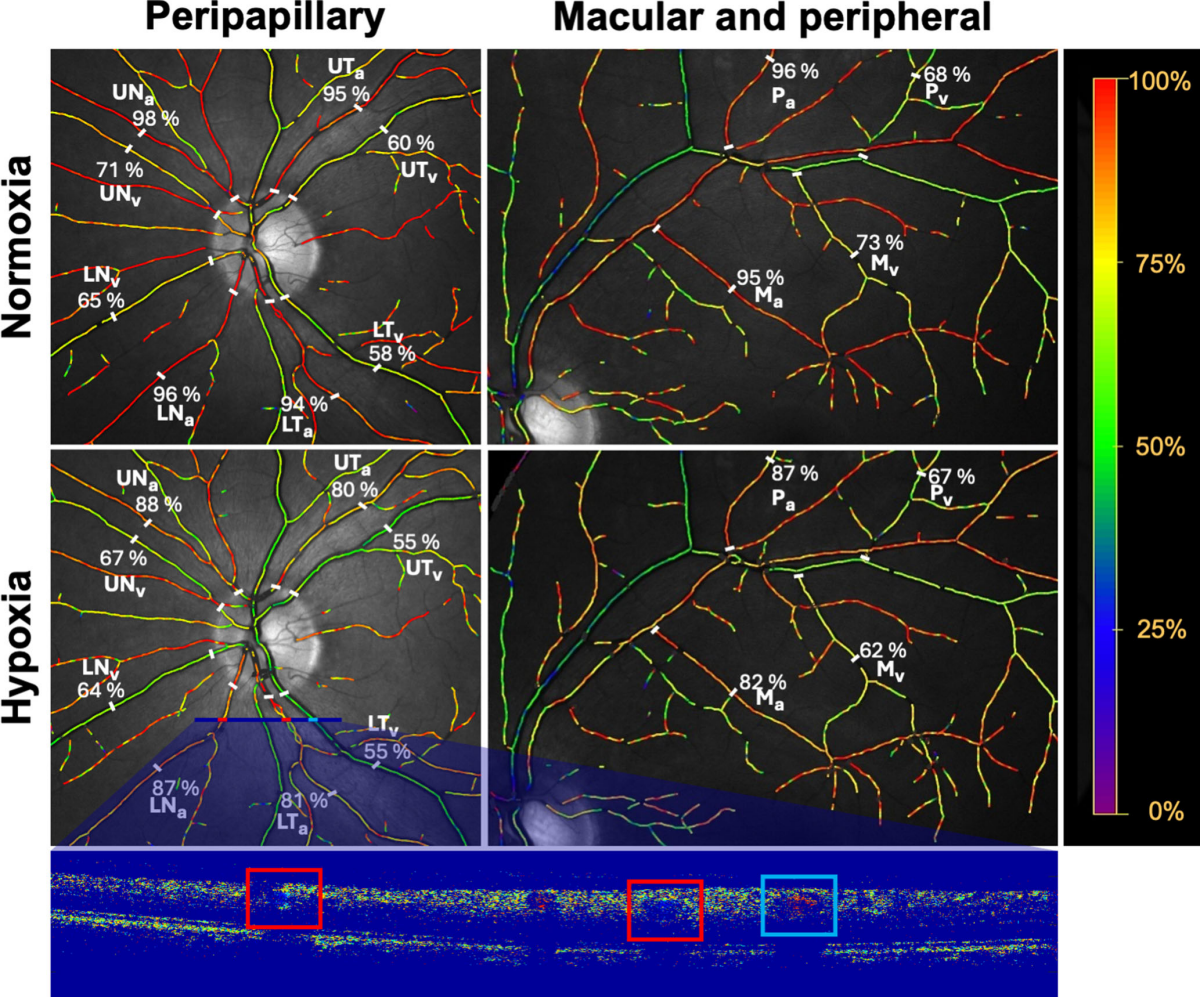
Oximetry images centered on the optic disc (*left*) showing the four selected peripapillary arterioles and venules and the upper temporal vessels (*right*) during normoxia and systemic hypoxia. The colors of the vessels represent the oxygen saturation as shown on the right vertical bar. The selected segments are delimited by *white bars* with the oxygen saturations shown at the end of each segment. UT, upper temporal; UN, upper nasal; LT, lower temporal; LN, lower nasal; P, peripheral; M, macular. Subscripts a and v denote arterioles and venules, respectively. The lower bar shows the Doppler OCT scan covering the lower nasal and temporal arterioles (*red rectangle*) and the lower temporal venule (*blue rectangle*).

Figure 2 shows that systemic hypoxia induced a significant reduction in the oxygen saturation in macular and peripheral arterioles as well as in macular venules (*P* < 0.003 for all comparisons), whereas no significant changes were observed in the peripheral venules (*P* = 0.95). The reduction in oxygen saturation was significantly lower in peripheral than in macular arterioles (*P* = 0.045) and venules (*P* = 0.036). It also appears that hypoxia significantly increased the diameter of both the peripheral and macular arterioles (*P* < 0.001 for both comparisons), but not the peripheral and macular venules (*P* > 0.08 for both comparisons). Finally, it appears that hypoxia significantly increased the linear velocity (*P* < 0.05 for all comparisons) and the blood flow (p < 0.04 for all comparisons) in all the studied branch vessels. There were no significant differences between these responses in peripheral and macular branch vessels (*P* > 0.35 for all comparisons). The responses in retinal oxygen saturations secondary to systemic hypoxia were so prominent that they could be observed directly in the color coding of retinal vessels in the oximetry images (Fig. 3).

## Discussion

The present study has shown how systemic hypoxia can affect the oxygen saturation, diameter, linear velocity and blood flow in peripapillary, peripheral, and macular arterioles and venules in normal persons. It is difficult to isolate experimental hypoxia to the eye without interfering with other parameters that influence ocular blood flow such as the intraocular pressure.^22^ Therefore retinal hypoxia was induced by breathing a hypoxic gas mixture. The limitation of this approach was that the observed effects on the retinal vessels might have been influenced by vascular responses outside the eye.

The examination program was extensive, and it was challenging for the participants to maintain a steady fixation while wearing the breathing system used to induce hypoxia. Therefore, after the initial measurement of the blood pressure, the sequence of events during normoxia was inverted during hypoxia so that all measurements of blood flow by Doppler OCT could be carried out without moving the participant. Resting periods were scheduled between all examination steps, and it was ensured that the duration of these periods were longer than the time required to stabilize the measured cardiovascular parameters.^23^^,^^24^ Therefore the results were considered not to be affected by carry over effects between the individual steps in the examinations.

Previous validations of the Doppler OCT system have included a confirmation that the total amount of blood measured to enter the retina through the arterioles is similar to the amount of blood leaving the retina through the corresponding venules.^16^^,^^25^ This was confirmed in the present study, although smaller arteriolar and venular branches crossing the disk border without connections to the larger vascular arcades were not included in the measurements. Moreover, the magnitude of the blood flow measured in the peripapillary vessels during both normoxia and hypoxia were consistent with previous findings.^26^^,^^27^ Measurements of the linear velocity of the blood obtained with the Doppler OCT technique are confined to phase shifts between −π and π generated by the moving blood cells. These limits may be exceeded with consequent erroneous results when linear velocities are high, causing the blood flow in the central part of the vessel to appear reversed.^15^^,^^28^ In the present study, this phenomenon was observed in only one vessel during normoxia and seven vessels during hypoxia. The exclusion of these measurements from the data material was considered not to have affected the results.

It has previously been shown that measurements of vessel diameters based on Doppler OCT scans tend to have a high variability because the shadow from the blood column interferes with the visibility of the lower vessel boundary.^15^ This artifact can result in an underestimation of the vessel diameter.^29^ Therefore the vessel diameters were calculated from the fundus photographs obtained by retinal oximetry using the vessel detection algorithm in the ARIA software.^20^ These calculations assumed an identical magnification in the optics of the eye in all individuals, which may not have been fulfilled with a consequent increase in the variability of the measured diameters and the derived estimates of blood flow. The separate measurements of diameters and the linear velocity of the blood used to calculate the blood flow may also have contributed to the variation in the measured diameter of the smaller vessel branches. This can explain that the observed dilation of peripheral and macular venules was not significant.

The higher oxygen saturation measured in peripheral than in macular arterioles during normoxia may be due to an underestimation of the oxygen saturation in vessels located in the lower half of the image as shown previously.^30^ However, because the location of the vessels was similar in the images obtained during normoxia and hypoxia, it is unlikely that this effect influenced the changes in oxygen saturation induced by hypoxia. Conversely, the effect emphasizes the validity of the observed higher oxygen saturation in macular than in peripheral venules. The study also confirmed that peripheral venules had a significantly larger diameter and blood flow than macular venules which has previously been shown to be balanced by a higher number of vessels due to a more extensive branching of macular than peripheral venules.^8^ This difference in the branching pattern can be assumed not to have affected the percentage change in vessel diameter and blood flow induced by hypoxia.

The oxygen saturation measured by retinal oximetry in peripapillary retinal arterioles in the present study was systematically lower than the saturation measured by finger oximetry which confirms findings from previous studies.^27^^,^^31^ Finger oximetry is considered to provide reliable measurements of the systemic arterial oxygen saturation.^32^ The measurement of a lower oxygen saturation by retinal oximetry may therefore be a consequence of how the oximeter software is calibrated to translate the relationship between the optical density of the light reflected from the vessels and the perivascular background at two different wavelengths into a value for the oxygen saturation.^31^ The finding may also be due to loss of oxygen by counter current exchange between the closely adjacent central retinal artery and vein in the optic nerve.^1^ However, the fact that the difference between the measured systemic and retinal oxygen saturations were maintained after the induction of systemic hypoxia, supports a systematic relation between the measured systemic and peripapillary oxygen saturations.

The breathing of the hypoxic gas mixture induced dilatation of the retinal arterioles. This is a normal response in metabolic autoregulation where the organism increases blood flow to compensate for an increase in oxygen consumption when metabolism is increased.^9^ The hypoxia-induced vasodilation in the systemic circulation was counteracted by an increase in the heart rate that resulted in a marginal increase in the arterial blood pressure. It is likely that this response had also elevated the driving pressure in the retinal arterioles as supported by the observed increase in the linear velocity of the blood in these vessels. However, it cannot be excluded that the response may also have been facilitated by a decrease in the vascular resistance in the retinal areas distal from where the diameter measurements were performed.

The reduction in the measured oxygen saturation in peripapillary retinal arterioles of approximately 11% (from 93.3% ± 3.7% to 82.5% ± 6.7%) during systemic hypoxia observed in the present study might be expected to be compensated by a similar increase in the delivery of oxygen and thereby blood flow. However, the blood flow increased by approximately 40% in both peripapillary arterioles (from 39.1 ± 12.9 µL/min to 53.7 ± 15.7 µL/min) and peripapillary venules (from 44.5 ± 12.2 µL/min to 65.6 ± 20.1 µL/min). If one assumes that the metabolic demand of the retina had remained unchanged^33^ and that all oxygen had been delivered by the retinal vascular system, the additional increase in blood flow would be expected to result in a proportionally lower reduction in the peripapillary A-V saturation difference. However, the reduction was only approximately 20%. This might be related to the variability in the measurements, but it could also be a consequence of a difference in the area supplied by the arterioles and drained by the adjoining venules during normoxia and hypoxia. Additionally, the finding might be secondary to increased linear velocity of the blood leading to a larger underestimation of the oxygen saturation in the arterioles than in the venules.^21^ Evidence suggests that systemic hypoxia can reduce the oxygen availability from the choroid and thereby increase oxygen extraction from the retinal vascular system^34^^–^^36^ that may contribute to explaining the results. Therefore it cannot be excluded that changes in the supply of oxygen from the choroid during hypoxia had affected oxygen saturations in the studied retinal venules. However, the finding of a higher increase in blood flow than would be expected from the decrease in oxygen is consistent with observations in the cerebral circulation where the observation has been suggested to be a consequence of limitations in the diffusion capacity of oxygen from the bloodstream to the neural tissue.^34^ This effect can be related to an increased mean transit time and to changes in the heterogeneity of the blood flow in the microcirculatory units.^37^

Regional variations in the extraction of oxygen from the retinal vascular system are of interest for understanding retinal vascular disease. It is therefore highly suggestive that the present study found a lower reduction in the oxygen saturation of arterioles supplying the periphery than the macular area. This might be due to differences in the rheology or oxygen extraction in the arteriolar blood reaching these branches. However, the study also showed no reduction in the oxygen saturation in the venules draining the retinal periphery during systemic hypoxia. In a previous study it was shown that an increase in the arterial blood pressure induced by isometric exercise can result in an increase in the oxygen saturation from retinal venules.^7^ The findings of the present study might be the result of a similar response if the hypoxia-induced increase in the retinal blood flow had limited the extraction of oxygen or increased the shunting of blood to bypass the peripheral microcirculation. This might point to the existence of a more general response pattern in the retinal vascular system where the regulation of the microcirculation and the consequent oxygen extraction differ among the peripheral and the central retina.^38^^,^^39^ The shunting of retinal blood to bypass the peripheral microcirculation might contribute to explaining that capillary occlusion predominates in this area in retinal disease.^2^

In conclusion, systemic hypoxia increases perfusion in the retina, but can affect the venous oxygen saturation in vessels draining the retinal periphery and the macular area differently. The findings might contribute to explaining regional variations in the clinical manifestations of retinal vascular disease.
